# Dynamic characteristics of *Neisseria meningitidis* carriage among new students in boarding martial arts schools: a longitudinal follow-up study

**DOI:** 10.3389/fpubh.2026.1941998

**Published:** 2026-10-22

**Authors:** Jun Li, Yingying Liu, Jiangnan Kong, Xiaoli Wang, Tian Tian, Xiangyang Zhang, Xiaoxiao Zhang, Yanfang Ji, Yonghao Guo, Qian Liu, Shuaiyin Chen, Yanyang Zhang

**Affiliations:** 1 Institute for Immunization Prevention and Programme, Henan Provincial Center for Disease Control and Prevention, Zhengzhou, China; 2 Henan Preventive Medicine Institute, Zhengzhou, China; 3 School of Public Health, Zhengzhou University, Zhengzhou, China; 4 Department of Immunization Program, Zhengzhou Municipal Center for Disease Control and Prevention, Zhengzhou, China; 5 Department of Immunization Program, Dengfeng Municipal Center for Disease Control and Prevention, Dengfeng, China

**Keywords:** boarding school students, carriage, hidden markov model, influencing factor, *Neisseria meningitidis*

## Abstract

**Objective:**

This study aimed to characterize the dynamic patterns of *Neisseria meningitidis* (Nm) carriage, identify factors influencing Nm acquisition and clearance, and provide empirical evidence for understanding the epidemiological mechanisms of Nm carriage and for developing targeted prevention strategies.

**Methods:**

A prospective cohort of 60 new entrants was established at a boarding martial arts school in Dengfeng City from February to December 2025. Oropharyngeal swabs were collected at regular intervals and subjected to laboratory testing. A hidden Markov model (HMM) was applied to estimate acquisition and clearance rates of Nm, and proportional hazards models were used to assess the effects of covariates on these rates.

**Results:**

A total of 13 follow-up visits and samplings were completed. The Nm carriage prevalence ranged from 30.00 to 93.75%, showing an overall upward trend, with serogroups B and X being predominant. During the observation period, 52.54% (31/59) of positive carriers exhibited ≥2 carriage episodes, and 35.59% (21/59) carried ≥2 serogroups over time. The mean carriage duration was estimated at 7.38 months (95% CI: 4.18–13.01). For Nm acquisition, individuals aged ≥15 years had a higher acquisition rate than those aged <15 years (HR = 2.01, 95% CI: 0.99–4.08), and those with a smoking history showed a higher acquisition rate than non-smokers (HR = 1.48, 95% CI: 0.76–2.91), although the latter difference was not statistically significant. For Nm clearance, age ≥15 years (HR = 4.40, 95% CI: 1.49–13.00) and fever in the past week (HR = 4.16, 95% CI: 1.36–12.75) were significantly associated with higher clearance rates. Smoking history (HR = 3.39, 95% CI: 0.92–12.53) and antibiotic use (HR = 2.58, 95% CI: 0.74–9.05) also suggested potential associations with increased clearance, but these did not reach statistical significance.

**Conclusion:**

New entrants in this boarding martial arts school exhibited a high risk of Nm carriage, with prolonged average carriage duration and frequent serogroup switching during carriage episodes. Age ≥15 years and recent fever were significantly associated with enhanced Nm clearance. These findings underscore the need for strengthened health surveillance and behavioral interventions to reduce the risk of persistent carriage and onward transmission in such congregate settings.

## 1 Introduction

*Neisseria meningitidis* (Nm) is a Gram-negative diplococcus that colonises the human nasopharyngeal mucosa. Upon breaching the mucosal barrier and invading the bloodstream, it can cause invasive meningococcal disease (IMD), which predominantly presents as meningococcal meningitis and septicaemia. Based on the structure and composition of the capsular polysaccharide, Nm is classified into 12 serogroups, of which six—A, B, C, W, X and Y—are the major causes of IMD worldwide (1). IMD is characterised by an abrupt onset, rapid progression, and high rates of disability and mortality. Even with timely and appropriate treatment, the case-fatality rate remains as high as 15% (2); moreover, 10–20% of survivors experience long-term sequelae, including hearing loss, limb amputation and neurological impairment, resulting in a considerable disease burden (3).

Nm is transmitted via respiratory droplets, primarily from asymptomatic carriers, as patients with invasive meningococcal disease are typically too ill to engage in activities that facilitate widespread community transmission. Furthermore, IMD cases are rarely linked between households, except in congregate settings such as college campuses, military barracks, and boarding schools, where close contact markedly enhances transmission. Carriage prevalence varies considerably by age; while overall estimates of 10–35% are commonly observed in adolescent and adult populations (4), Asymptomatic carriers are difficult to identify, and carriage can persist for 5 to 9 months (5, 6). In Henan Province, the incidence of meningococcal disease declined dramatically from 64.71 per 100,000 during the natural epidemic period (1950–1979) to 0.01 per 100,000 in the expanded immunisation era (2008–2021). Nevertheless, sporadic outbreaks continue to occur locally (7). In 2017, a cluster of IMD cases was reported in a boarding martial arts school in Dengfeng City, Henan Province. Subsequent carriage surveys among healthy students in this school revealed a prevalence of 31.19–45.83% (8), indicating a substantially elevated risk in this congregate setting. In light of these observations, we established a prospective cohort study with repeated follow-up sampling to systematically investigate the dynamic patterns of Nm carriage and its associated factors. The findings are expected to advance our understanding of the natural history of Nm colonisation and to inform evidence-based strategies for the prevention and control of meningococcal disease.

## 2 Methods

### 2.1 Study participants

The study was conducted at a boarding martial arts school in Dengfeng City, Henan Province, China. This type of school operates on a full-time residential basis, with students living in shared dormitories, eating in communal canteens, and participating in daily routines that combine academic courses with intensive martial arts training. The training involves frequent close physical contact and prolonged indoor crowding, and students are recruited from various regions across China, contributing to high population mobility and potential introduction of diverse Nm strains. A follow-up study was conducted at a boarding martial arts school in Dengfeng City, Zhengzhou, from February to December 2025. Two classes were randomly selected from newly enrolled students using cluster sampling. Inclusion criteria were: (1) new students enrolled in spring 2025; (2) students who themselves or whose guardians agreed to undergo sample collection, survey, and follow-up after enrollment; and (3) students expected to complete long-term follow-up sampling and surveys. Exclusion criteria were: (1) previously enrolled students; (2) those with known immune system diseases or who were in the acute phase of any illness. Written informed consent was obtained from all participants at the time of enrollment. For participants under 18 years of age, written informed consent was obtained from their parents or legal guardians prior to enrollment. All consent procedures were approved by the Ethics Committee of Henan Provincial Center for Disease Control and Prevention.

### 2.2 Field investigation and laboratory sampling

Thirteen follow-up visits were conducted at enrollment/1 week/2 weeks/3 weeks/4 weeks/5 weeks/6 weeks/7 weeks/9 weeks/11 weeks/22 weeks/27 weeks/42 weeks. Oropharyngeal swabs were collected by trained professionals from the Dengfeng Center for Disease Control and Prevention. Sterile oropharyngeal swabs (Yocon Hengde Biotechnology (Beijing) Co., Ltd., model BZ0304, lot number Y71240902) were used. The swab was passed beyond the base of the tongue, rubbed firmly back and forth at least 3 times on both tonsils, and then rubbed up and down at least 3 times on the posterior pharyngeal wall. After collection, the swab was immediately inoculated onto chocolate agar plates (Guangdong Huankai Microbial Sci. & Tech. Co., Ltd., product number 029031) using a three-sector streaking method. The plates were temporarily stored in an insulated box containing warm water bags and transported to the laboratory within an average of 3 h. Subsequently, the plates were incubated at 37 °C with 5% CO2 for 18–24 h. Basic characteristics of the study participants, including age, sex, vaccination history, smoking history, and training duration, were collected through questionnaires. At each follow-up visit, information on fever (axillary temperature ≥37.3 °C) and antibiotic use within the past week, changes in dormitory assignment, and vaccination status was also collected.

### 2.3 Laboratory testing

1. *Bacterial culture and identification*: Colonies with typical morphology of *Neisseria meningitidis* (circular, moist, convex, glistening, translucent grey-white colonies) were picked from chocolate agar plates and subcultured for purification. Gram staining was then performed, and Gram-negative kidney-shaped diplococci were identified by microscopy. Oxidase testing was subsequently conducted, and oxidase-positive strains were further subjected to biochemical identification. Strains that fermented glucose and maltose with acid production but no gas formation were preliminarily identified as Nm.
2. *Nucleic acid extraction*: Bacterial genomic DNA was extracted using a commercial bacterial genomic DNA extraction kit (Xi’an Tianlong Science and Technology Co., Ltd., lot number 24121210 T032) on a fully automated nucleic acid extraction instrument according to the manufacturer’s instructions. In each batch, extraction blank controls and positive controls were included in accordance with the laboratory standard operating procedures.
3. *Real-time fluorescence PCR*: Qualitative detection of *N. meningitidis* was performed using an Nm ctrA/sodC duplex real-time fluorescence PCR kit (Beijing Zhuocheng Huisheng Biotechnology Co., Ltd., catalog number A7192YH-50 T, lot number F202411022). Nm-positive samples were further serogrouped using two triplex real-time fluorescence PCR kits: one for serogroups A/B/C (catalog number A5933YH-50 T, lot number T202412005) and another for serogroups W/X/Y (catalog number A5943YH-50 T, lot number H202412250).

The PCR master mix components, total reaction volume, and sample input volume were prepared according to the manufacturer’s instructions. No-template controls (NTCs) and extraction blank controls were included in each PCR batch. The PCR amplification program consisted of an initial denaturation/polymerase activation step at 95 °C for 30 s (1 cycle), followed by 40 cycles of denaturation at 95 °C for 5 s, and annealing/extension at 60 °C for 30 s, with fluorescence signal acquisition during the 60 °C annealing/extension step.

*Instrument model*: A fully automated real-time fluorescence PCR instrument with multi-channel calibration (equipped with FAM, VIC(HEX), and CY5 detection channels) was used, such as ABI7500, 7500FAST, Bio-Rad CFX96, Roche LightCycler 480II, Shanghai Hongshi SLAN-96P, etc.

*Correspondence between fluorescent channels and targets*: For kit A7192, the FAM channel detects the ctrA gene, and the VIC (HEX) channel detects the sodC gene. For kit A5933, the FAM, VIC (HEX), and CY5 channels detect serogroups A, B, and C, respectively. For kit A5943, the FAM, VIC (HEX), and CY5 channels detect serogroups X, Y, and W135, respectively.

*Internal amplification control and assay validity*: The kits contain negative and positive controls containing positive templates for each corresponding detection target. An assay run was considered valid only if no amplification curve was observed for the negative control and a typical S-shaped amplification curve was obtained for the positive control in the corresponding detection channel. If the NTC or extraction blank control showed positive amplification, the entire batch was deemed invalid, and all samples were re-extracted and retested.

*Result interpretation*: The cutoff Ct value for a positive result was ≤ 35; the grey zone (borderline range) was defined as 35 < Ct ≤ 40. Strains positive for Nm species-specific targets (ctrA/sodC) but negative for all tested serogroup-specific targets (A, B, C, X, Y, and W135) were defined as non-groupable (NG) strains. According to the kit’s instructions, for oropharyngeal swab samples, a positive result for sodC alone with a negative ctrA result cannot confirm Nm infection (it may indicate other pathogens or non-capsular strains). Therefore, ctrA positivity was strictly used as the diagnostic criterion for Nm in this study, and samples with sodC single-positivity were excluded during the review stage.

### 2.4 Statistical analysis

The hidden Markov model is a probabilistic model for time-series data that describes the process of generating an unobservable state sequence from a hidden Markov chain, with each state generating an observation to produce an observable sequence. The sequence of states generated by the hidden Markov chain is referred to as the state sequence, while the observable sequence generated from each state is referred to as the observation sequence. The HMM is defined by three elements: the initial probability distribution π, the state transition probability distribution A, and the observation probability distribution B (9). Data were collated and summarized using Excel 2019. Statistical analyses and graphing were performed using R version 4.5.0. The significance level α was set at 0.05.

#### 2.4.1 Model parameters

A continuous-time hidden Markov model was employed to quantify individual-level Nm carriage dynamics within a Susceptible-Infected-Susceptible (SIS) framework (10–12), as illustrated in Figure 1. The HMM comprised two unobservable hidden states: the susceptible state (S) and the infected state (I). The transition probability matrix was defined as A (t)= $\left[\begin{array}{cc} 1-a_{12} & a_{12}\\ a_{21} & 1-a_{21} \end{array}\right]$ , where $a_{12}$ represents the Nm acquisition rate and $a_{21}$ represents the Nm clearance rate (13, 14). In the HMM, the state sequence (X*_i_*) for each individual *i* at time *t* is not directly observable; instead, it is approximated by the observation sequence (Y*i*) obtained from pharyngeal swab test results (negative or positive) (15–17). Accordingly, the observation matrix was defined as B= $\left[\begin{array}{cc} P_{r}(Y=0|X=S) & P_{r}(Y=1|X=S)\\ P_{r}(Y=0|X=I) & P_{r}(Y=1|X=I) \end{array}\right]$ = $\left[\begin{array}{cc} 1-b_{12} & b_{12}\\ b_{21} & 1-b_{21} \end{array}\right]$ , where $b_{12}$ is the false-positive rate and b_21_ is the false-negative rate. Assuming 100% specificity of PCR testing, the observation matrix was simplified to B= $\left[\begin{array}{cc} 1 & 0\\ b_{21} & 1-b_{21} \end{array}\right]$ . To evaluate the effects of covariates on Nm acquisition and clearance, we fitted proportional hazards models to the transition intensities (11). Specifically, the acquisition intensity from the susceptible state (S) to the infected state (I) for individual i at time *t* was modelled as $q_{12}(z_{i}(t))=q_{12}^{\left(0\right)}\exp(\beta_{12}^{T}z_{i}(t))$ , and the clearance intensity from I to S as $q_{21}(z_{i}(t))=q_{21}^{\left(0\right)}\exp(\beta_{21}^{T}z_{i}(t))$ , where $z_{i}(t)$ is the covariate vector, $q_{12}^{\left(0\right)}$ and $q_{21}^{\left(0\right)}$ are the baseline transition intensities, and exp.( $\beta^{T}z_{i}(t)$ ) represents the hazard ratio associated with a unit change in the corresponding covariate. Maximum likelihood estimation was performed using the msm package in R with the BOBYQA optimisation algorithm (13, 18).

**Figure 1:**
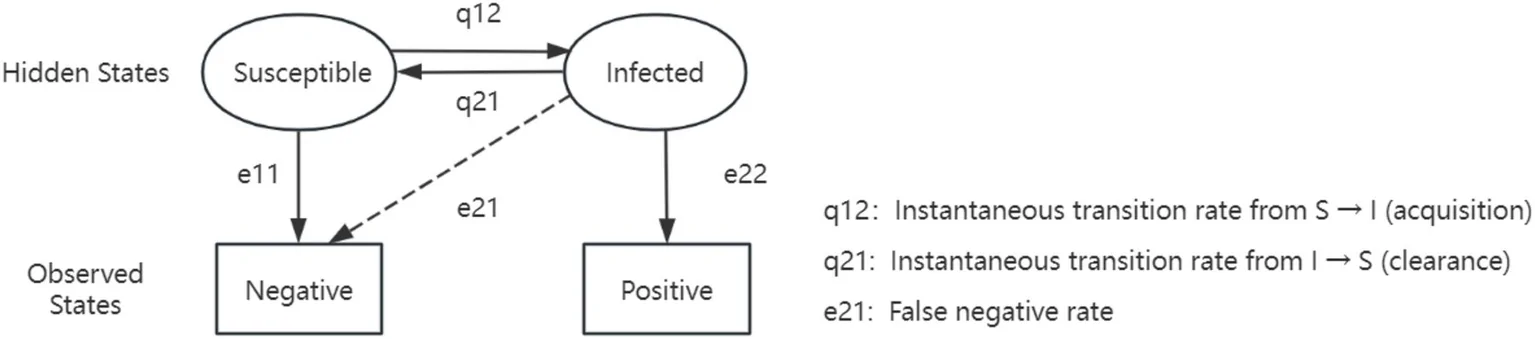
Schematic diagram of the Susceptible–Infected–Susceptible (SIS) hidden Markov model for Nm carriage dynamics.

#### 2.4.2 Model evaluation

1. *Model selection*: To assess the robustness of the primary model results, we constructed three additional alternative models and compared their fitting performance with that of the primary model. Model fit was evaluated using the Akaike Information Criterion (AIC), and we also examined whether the conclusions drawn from the different models differed substantively.
2. *Convergence assessment*: Five different sets of initial transition matrices were used to fit the model separately, with each run iterating at least 1,000 times. If the resulting transition probability estimates and the −2 log-likelihood values were similar across the five repeated fittings, the model was considered to have converged stably, indicating reliable parameter estimation (11, 12).
3. *Goodness-of-fit evaluation*: The Viterbi algorithm was applied to decode the most probable sequence of hidden states for each individual at each time point. Both the observed data and the model-predicted carriage statuses were grouped into 14-day intervals, and the Nm carriage prevalence was calculated for each interval. The observed prevalence values were then compared with the model-predicted values along with their 95% confidence intervals. If the observed values fell within the predicted intervals and the overall trends were consistent, the model was considered to adequately capture the underlying transmission dynamics of Nm (11).
4. *Sensitivity analysis*: To examine the impact of the false-negative rate on the results, we set the false-negative rate to 2%, 5%, and 10% respectively, fixed the observation probability matrix, and refitted the model. The weekly acquisition and clearance rates of Nm and the covariate effects were compared across these scenarios. If the key estimates maintained consistent directions and showed only minor numerical fluctuations, the primary model results were considered robust and reliable.

#### 2.4.3 Handling of missing data and loss to follow-up

During follow-up, some participants were lost to follow-up or missed individual visits owing to school transfers, dropouts, or examinations, resulting in intermittent missing data in the longitudinal sequence. For missing covariates, multiple imputation was performed using the mice package in R. An imputation model was constructed using chained equations, with individual ID, follow-up time, baseline demographic characteristics, time-varying exposures, and the observed Nm carriage status as predictors. Five complete datasets were generated, and convergence of the imputation procedure was verified; the results were pooled across datasets using Rubin’s rules. For missing Nm carriage status (the outcome), the hidden Markov model (HMM) inherently accommodates missing outcome data within its likelihood framework. Specifically, the forward–backward algorithm and the expectation–maximization (EM) algorithm, used for parameter estimation and state decoding, directly integrate over the missing observations, assuming missingness at random conditional on the observed data. Together with multiple imputation for covariates, this approach maximised the use of all available information and minimised potential bias introduced by loss to follow-up and data gaps.

## 3 Results

### 3.1 Baseline demographic characteristics

A total of 60 participants were enrolled in the cohort, all of whom were male (due to the low number of female students at the martial arts school). Age ranged from 13 to 18 years, with a median of 14 years (IQR: 13–15). Among them, 56.67% (34/60) were aged <15 years, and 43.33% (26/60) were aged ≥15 years. A total of 66.67% (40/60) had a history of smoking, whereas 33.33% (20/60) had no smoking history. In terms of dormitory density, 41.67% (25/60) had a per capita dormitory area of <3 m^2^, and 58.33% (35/60) had a per capita dormitory area of ≥3 m^2^. The 60 participants were divided into two classes: Sanda and Taolu. The per capita classroom area was 2.2 m^2^ (65 m^2^/30 students) in the Sanda class and 1.3 m^2^ (65 m^2^/50 students) in the Taolu class. A total of 11.67% (7/60) reported a history of fever in the past week, while 88.33% (53/60) did not. All participants had a history of vaccination with the ACYW135 group meningococcal polysaccharide vaccine, of whom 96.67% (58/60) received the vaccine either before enrollment or within one month after enrollment. Detailed baseline characteristics are presented in Table 1.

**Table 1:** Baseline characteristics of the cohort of new students.

Variable	Category	Participants (*n*)	Positive cases (*n*)	Carriage rate (%)
Sex	Male	60	18	30.00
	Female	0	0	0.00
Age (years)	<15	34	8	23.53
	≥15	26	10	38.46
Smoking history	Yes	40	15	37.50
	No	20	3	15.00
Dormitory per capita area (m^2^)	<3	25	8	32.00
	≥3	35	10	28.57
Classroom per capita area (m^2^)	1.3	30	7	23.33
	2.2	30	11	36.67
Fever in the past week	Yes	7	2	28.57
	No	53	16	30.19

### 3.2 Carriage rate

A total of 13 surveys and sample collections were completed to monitor the Nm carriage rate in the cohort of new students at the martial arts school, yielding 703 pharyngeal swab specimens. The carriage rate at enrollment was 30.0%, and over the subsequent 12 sampling time points, the carriage rate ranged from 35.00 to 93.75%, showing an overall upward trend. Serogroups B and X were the predominant strains. Serogroup A was not detected. The proportion of serogroup B ranged from 32.26 to 70.37%, serogroup C from 4.17 to 16.67%, serogroup X from 9.52 to 47.73%, serogroup Y from 4.55 to 16.67%, serogroup W135 from 0 to 6.98%, and non-groupable strains from 0 to 2.33%. The carriage rate at each follow-up visit is shown in Table 2.

**Table 2:** Carriage prevalence and serogroup distribution of *Neisseria meningitidis* among new students at a martial arts school from February to December 2025.

Sampling date	Carriage prevalence (%)	B	C	X	Y	W135	Non-groupable
Feb.18	30.00 (18/60)	66.67 (12/18)	5.56 (1/18)	11.11 (2/18)	16.67 (3/18)	0	0
Feb.25	35.00 (21/60)	66.67 (14/21)	9.52 (2/21)	9.52 (2/21)	14.29 (3/21)	0	0
Mar.4	45.00 (27/60)	70.37 (19/27)	7.41 (2/27)	11.11 (3/27)	11.11 (3/27)	0	0
Mar.11	76.67 (46/60)	47.83 (22/46)	6.52 (3/46)	34.78 (16/46)	6.52 (3/46)	4.35 (2/46)	0
Mar.18	83.33 (50/60)	44.00 (22/50)	6.00 (3/50)	38.00 (19/50)	6.00 (3/50)	4.00 (2/50)	2.00 (1/50)
Mar.26	74.58 (44/59)	40.91 (18/44)	4.55 (2/44)	47.73 (21/44)	4.55 (2/44)	2.27 (1/44)	0
Apr.1	74.14 (43/58)	39.53 (17/43)	4.65 (2/43)	44.19 (19/43)	4.65 (2/43)	4.65 (2/43)	2.33 (1/43)
Apr.8	81.36 (48/59)	41.67 (20/48)	4.17 (2/48)	41.67 (20/48)	6.25 (3/48)	6.25 (3/48)	0
Apr.21	85.45 (47/55)	36.17 (17/47)	6.38 (3/47)	44.68 (21/47)	6.38 (3/47)	6.38 (3/47)	0
May.6	81.13 (43/53)	39.53 (17/43)	4.65 (2/43)	44.19 (19/43)	4.65 (2/43)	6.98 (3/43)	0
Jul.21	73.81 (31/42)	32.26 (10/31)	6.45 (2/31)	45.16 (14/31)	9.68 (3/31)	6.45 (2/31)	0
Aug.25	82.22 (37/45)	32.43 (12/37)	10.81 (4/37)	43.24 (16/37)	8.11 (3/37)	5.41 (2/37)	0
Dec.8	93.75 (30/32)	33.33 (10/30)	16.67 (5/30)	40.00 (12/30)	6.67 (2/30)	3.33 (1/30)	0

### 3.3 Carriage acquisition rate and duration

Of the 703 pharyngeal swab specimens, 485 tested positive, yielding a positivity rate of 68.99%. Over the 13 follow-up visits, 98.33% (59/60) of participants had at least one positive test result. Among the positive carriers, 52.54% (31/59) had ≥2 carriage episodes, and 35.59% (21/59) carried ≥2 distinct serogroups. During the 10-month follow-up period, the longest observed carriage duration was ≥9 months for serogroup B and X, ≥4 months for serogroup C, ≥8 months for serogroup W135 and Y. Changes in carriage status over time for all participants are shown in Figure 2. Among the 485 positive samples, 483 (99.59%) were double-positive for both ctrA and sodC, and 2 (0.41%) were ctrA single-positive with sodC negative. No sodC single-positive samples were recorded. The double-positive agreement rate was 99.59%, indicating excellent consistency between the two target genes.

**Figure 2:**
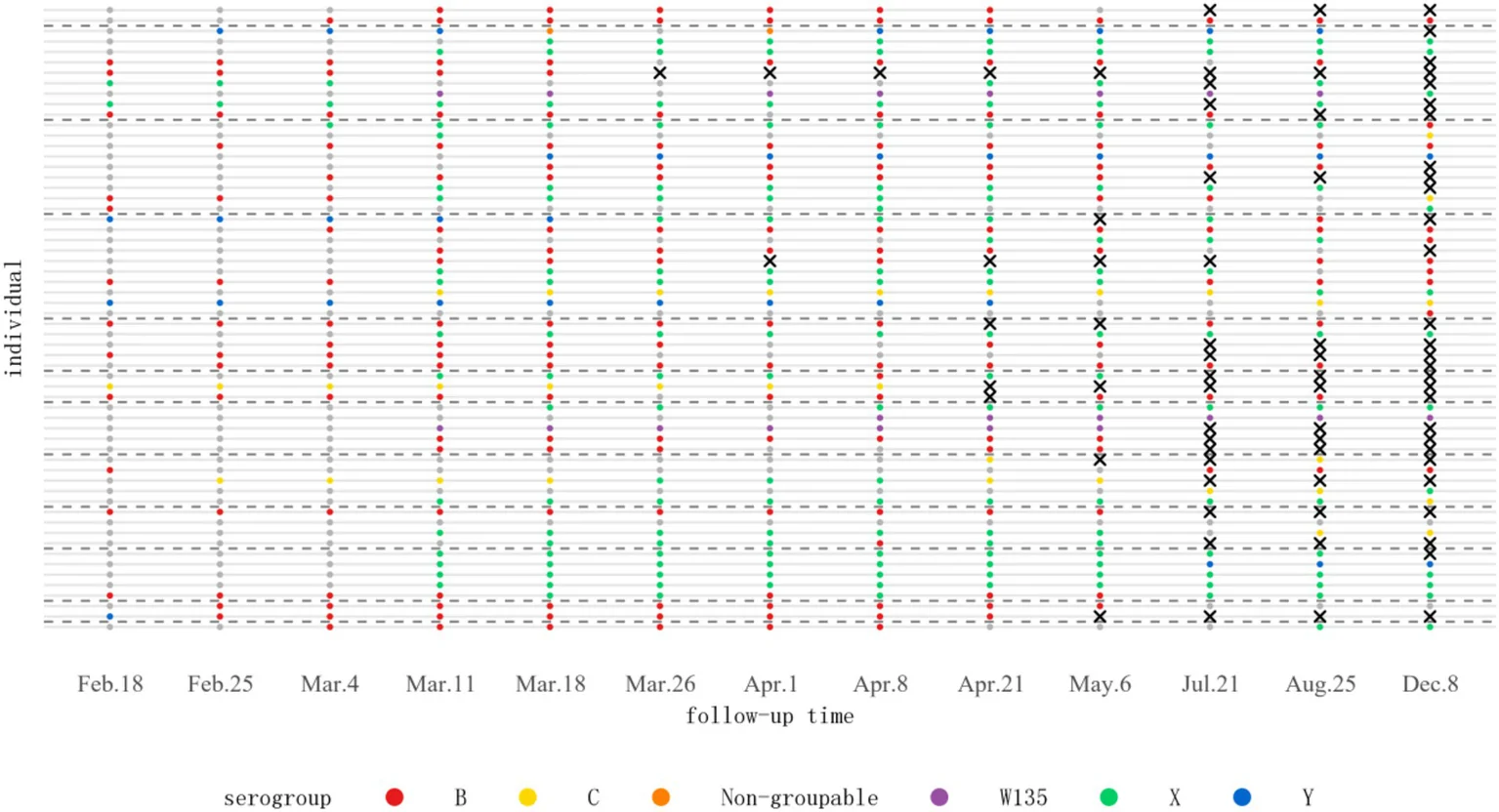
Distribution of serogroup carriage status during follow-up among participants from different dormitories (Each solid line represents one participant; dashed lines separate participants by dormitory; ‘×’ indicates missing sampling due to loss to follow-up or other reasons).

Based on the continuous-time hidden Markov model, the overall weekly Nm acquisition rate was 21.77% (95%CI: 15.75–30.10%), and the overall weekly clearance rate was 3.16% (95%CI: 1.79–5.58%), corresponding to a mean carriage duration of 7.38 months (95%CI: 4.18–13.01). The estimated probability of a false-negative pharyngeal swab result was 1.43% (95%CI: 0.44–4.55%).

### 3.4 Factors associated with nm acquisition and clearance

The best-fitting model included age, smoking history, and dormitory floor area per person as covariates for Nm acquisition, and age, smoking history, fever history, and antibiotic use as covariates for Nm clearance. For acquisition, individuals aged ≥15 years had a higher rate than those aged <15 years (HR = 2.01, 95% CI: 0.99–4.08); those with a smoking history showed a higher acquisition rate than non-smokers (HR = 1.48, 95% CI: 0.76–2.91); and participants with a dormitory area of <3 m^2^ per person had a lower acquisition rate compared with those with ≥3 m^2^ per person (HR = 0.79, 95% CI: 0.40–1.55). However, none of these associations reached statistical significance.

For clearance, age ≥15 years (HR = 4.40, 95% CI: 1.49–13.00) and fever within the past week (HR = 4.16, 95% CI: 1.36–12.75) were significantly associated with higher Nm clearance rates. Smoking history (HR = 3.39, 95% CI: 0.92–12.53) and antibiotic use (HR = 2.58, 95% CI: 0.74–9.05) also showed trends toward increased clearance, although these associations did not reach statistical significance (Figure 3).

**Figure 3:**
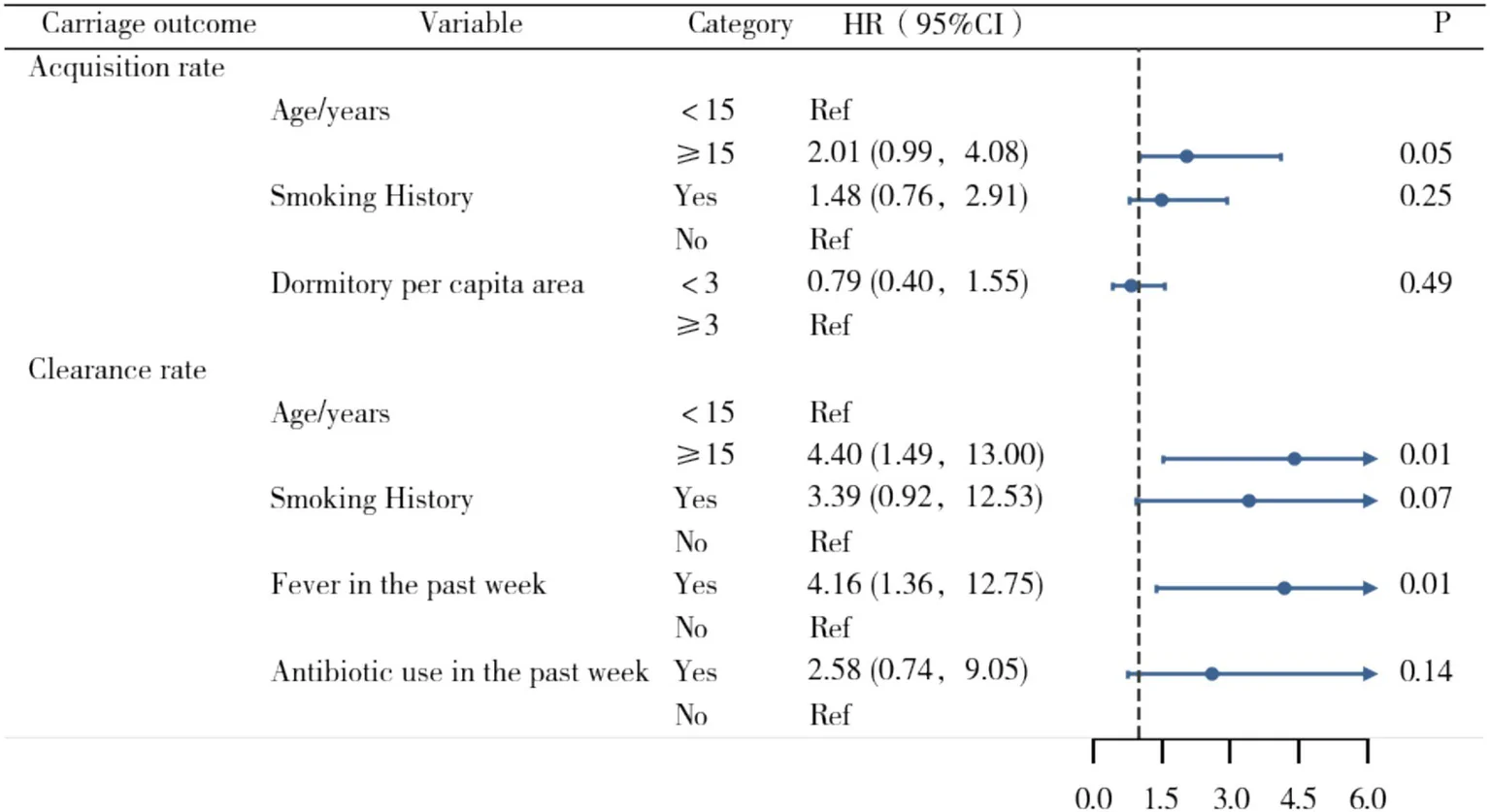
Covariate effects on Nm acquisition and clearance rates.

### 3.5 Model evaluation

Comparison of models showed that the model including age, smoking history, and dormitory per capita area as covariates for the Nm acquisition rate, and age, smoking history, history of fever, and history of antibiotic use as covariates for the Nm clearance rate yielded the lowest AIC score, indicating the best model fit (Table 3). For this optimal model, five different initial transition intensities were selected, and five Markov chains were run. All chains converged to similar transition intensity levels, with minimal variation in the −2 log-likelihood values, indicating model convergence and robust parameter estimation (Figure 4). Comparison of observed data with model-predicted carriage status showed that the observed values generally fell within the 95% confidence intervals of the predicted values, and the overall trends of the two series were highly consistent, suggesting that the constructed model could realistically simulate the dynamic transmission patterns of Nm (Figure 5). Sensitivity analyses, performed by fixing the false-negative rate at 2%, 5%, and 10%, showed that the estimated weekly acquisition rates ranged from 20.80 to 21.34%, and the weekly clearance rates ranged from 1.84 to 3.16%. Across all scenarios, the direction of covariate effects remained largely consistent, and the confidence intervals overlapped substantially with those derived from the primary model (Figure 6).

**Table 3:** Model comparison results based on AIC.

Model	Covariates for Nm acquisition rate	Covariates for Nm clearance rate	AIC	−2Log-likelihood
1	Age + Smoking + Fever + Antibiotic + Dormitory area + Classroom area	Age + Smoking + Fever + Antibiotic + Dormitory area + Classroom area	531.60	499.60
2	Age + Smoking + Fever + Dormitory area + Classroom area	Age + Smoking + Fever + Antibiotic + Dormitory area + Classroom area	529.75	499.75
3	Age + Smoking + Dormitory area	Age + Smoking + Antibiotic	527.55	507.55
4	Age + Smoking + Dormitory area	Age + Smoking + Fever + Antibiotic	524.67	502.67

**Figure 4:**
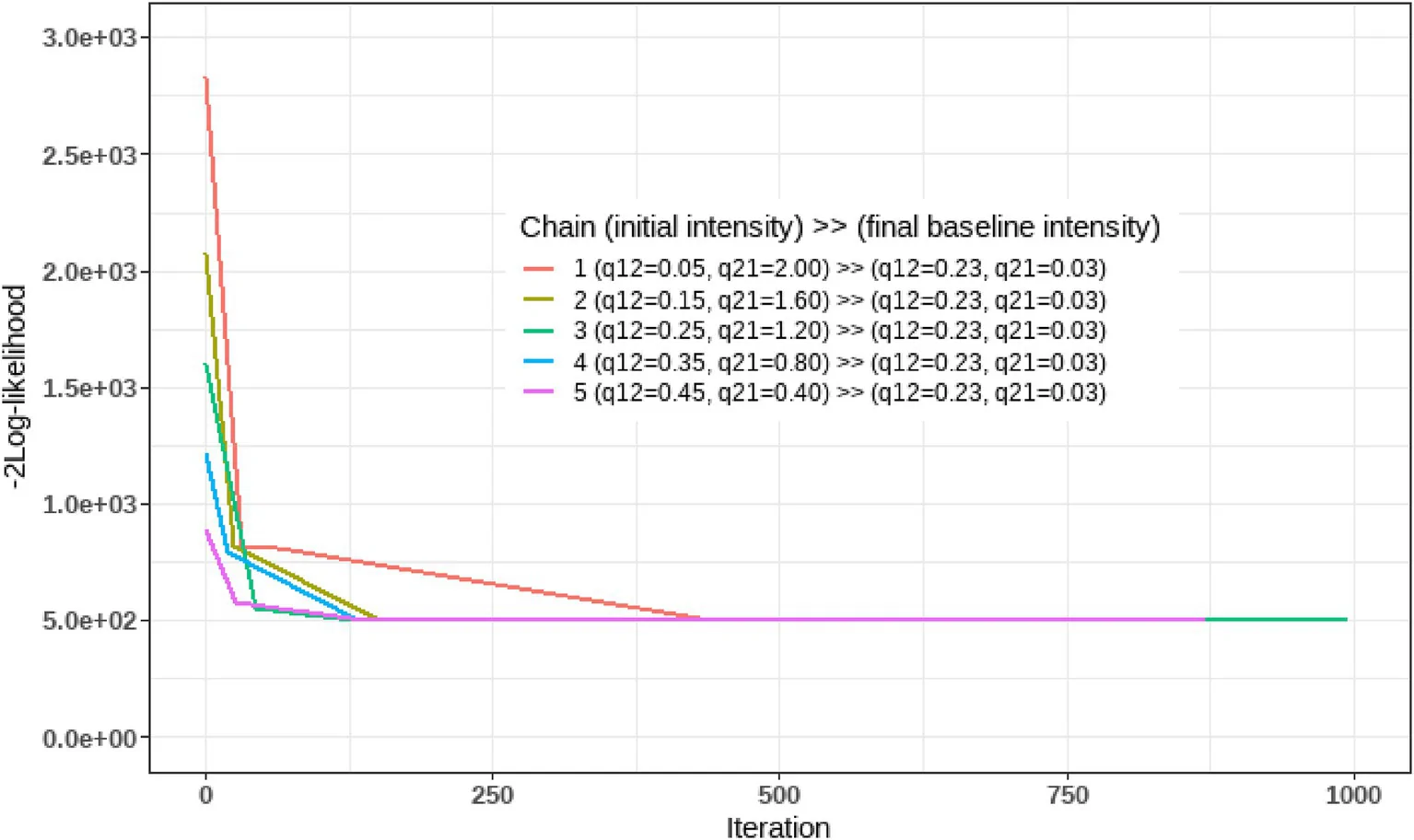
Results of convergence assessment.

**Figure 5:**
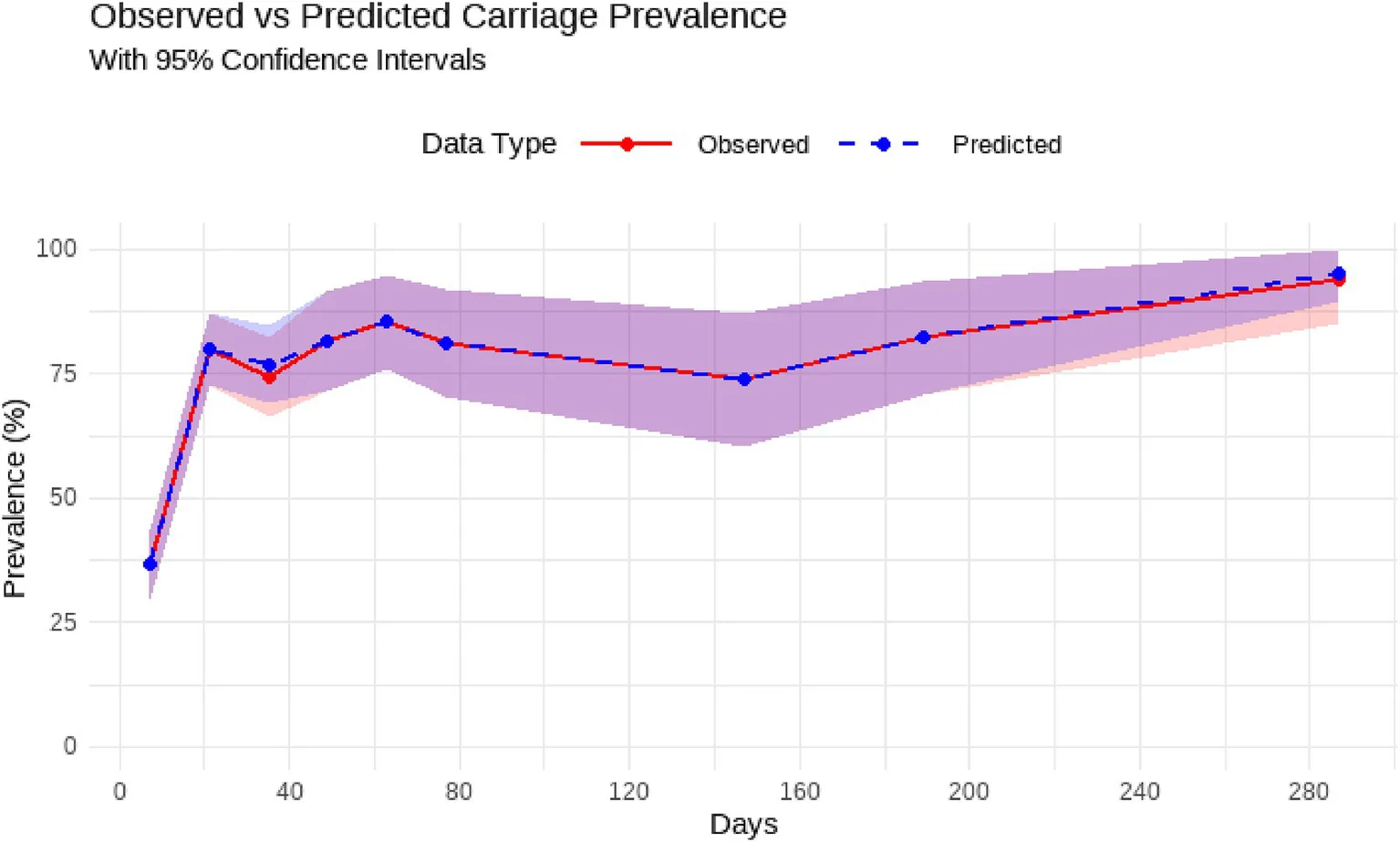
Comparison between observed carriage rates and model-predicted values (the red solid line with dots represents the observed carriage prevalence; the blue dashed line represents the model-predicted carriage prevalence; the purple shaded area represents the 95% confidence intervals (CI) of the predicted values; and the orange shaded area represents the 95% CI of the observed values).

**Figure 6:**
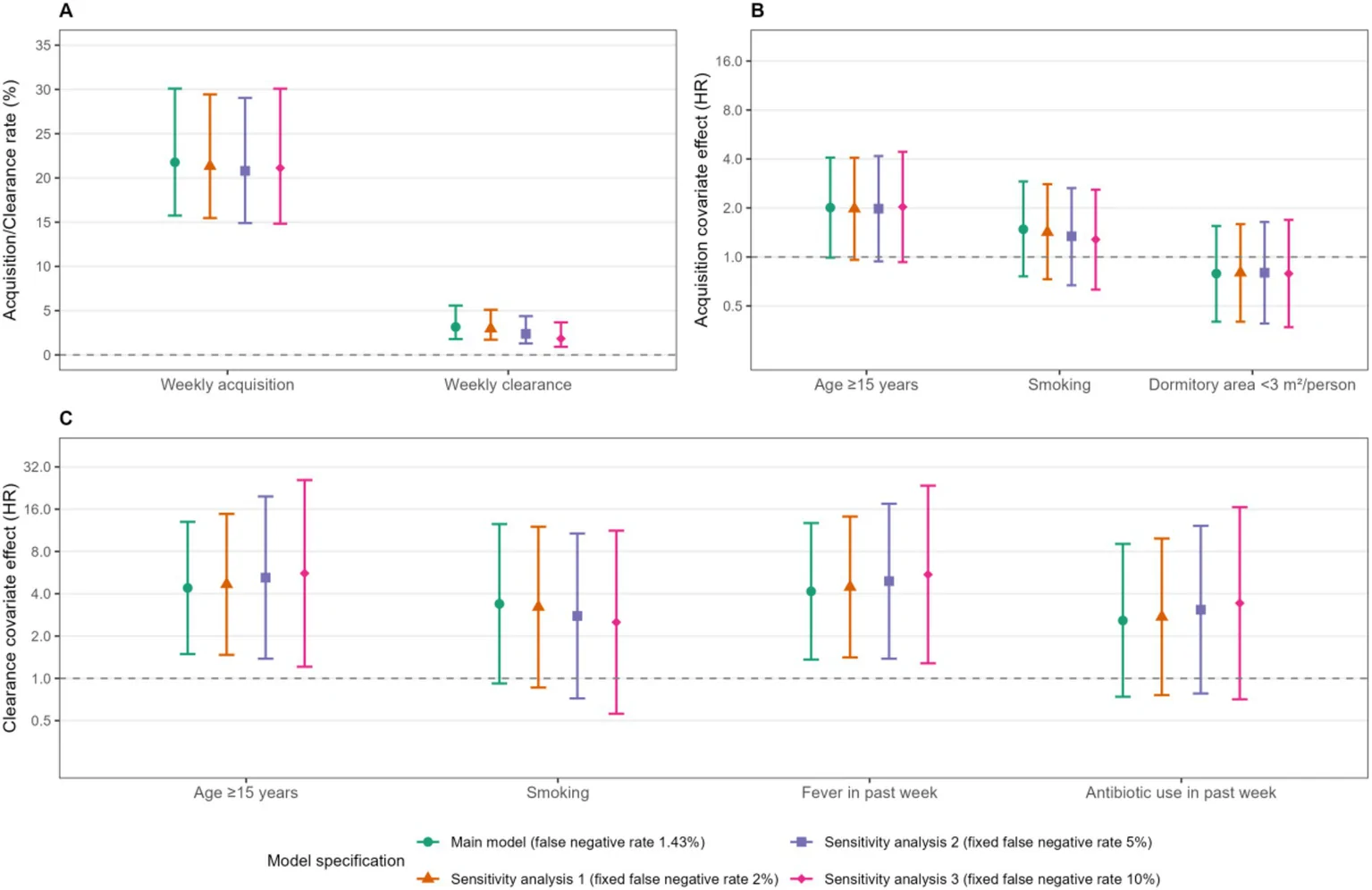
Sensitivity analyses of model parameters to varying false-negative rates. **(A)** Weekly acquisition and clearance rates of *N. meningitidis*; **(B)** Covariate effects on acquisition; **(C)** Covariate effects on clearance. Reference categories for all covariates were: age <15 years, non-smoking, dormitory floor area ≥3 m^2^ per person, no fever in the past week, and no antibiotic use.

## 4 Discussion

Healthy carriers are the primary source of Nm transmission. Due to crowded living environments and frequent close contact, boarding school students have become a high-risk population for Nm carriage and transmission. Currently, domestic studies on Nm carriage have mainly focused on cross-sectional surveys. Although these studies have preliminarily explored the risk factors associated with carriage, there remains a lack of in-depth research on the dynamic patterns of Nm carriage evolution and the associated risk factors.

In this 10-month longitudinal follow-up study, the carriage rate among 60 new students at a boarding martial arts school increased from 30.0% at enrollment to 83.33% after one month, remained at a high level thereafter, and gradually reached 93.75% after 10 months. This trend is similar to the findings of previous studies. A study conducted in 2018 at a martial arts school in Dengfeng City reported that the carriage rate among new students increased from 13.27% at enrollment to 30.09% after 3 months (19). In a longitudinal study in the United Kingdom, the Nm carriage rate among university students rose from 13.9% in the first week to 34.2% after 2 months (20). These findings collectively indicate that the risk of Nm carriage is elevated during the early period after school entry. Therefore, health education programs targeting this high-risk population should be implemented to raise awareness of disease transmission risks, and enhanced entry screening and dynamic surveillance during the school year are warranted to effectively identify potential transmission risks. The Nm carriage rate observed in the present cohort of martial arts school new students was considerably higher than those reported in longitudinal studies among Australian adolescents (2.0–7.7%) (21), Canadian university students (19.1–24.2%) (22), and first-year university students in the United Kingdom (47–62%) (6), as well as the carriage rate reported in a study at a high school in Suizhou City, China (27.12–40.28%) (23). The students at the martial arts school came from diverse geographic origins and exhibited high mobility. The curriculum consisted of half-day academic classes and half-day martial arts training, during which students frequently engaged in close-contact activities. These factors may collectively facilitate Nm transmission. Moreover, the predominant serogroups in this study were B and X, which differs from the results of a previous cross-sectional study conducted at the same boarding martial arts school (where serogroups B and C predominated), suggesting a diversification of circulating strains across different regions and populations. Currently, serogroup B Nm is gradually becoming the main epidemic serogroup in China, and cases caused by serogroups X, W, and Y are also showing an increasing trend (24). Internationally, vaccines against serogroup B Nm have been successfully developed and deployed, which can effectively prevent serogroup B IMD (25). Furthermore, multivalent combination vaccines have become a global trend in vaccine development to effectively address the diversified threat of meningococcal serogroups.

The results of this study showed that the vast majority of participants tested positive for Nm at least once during the observation period, with an overall sample positivity rate of 68.99%, which was significantly higher than the carriage rates reported in a study from Quebec, Canada (8.8–29.1%) (22). During the observation period, more than half of the participants experienced ≥2 carriage episodes, and 35.59% of individuals carried ≥2 different serogroups. These frequencies were generally consistent with the rates of strain replacement among persistent carriers reported in studies from South Australia (24.0%) (21) and the University of Nottingham, United Kingdom (36.4%) (6). These data suggest that the study population lived in an environment with highly active Nm transmission, where carriage status fluctuated frequently over time. Moreover, the immune protection acquired against one serogroup may not be sufficient to prevent colonization by other serogroups, a finding consistent with the serogroup-specific nature of immune protection against *Neisseria meningitidis* (26). In this study, the estimated mean duration of Nm carriage among boarding martial arts school students was 7.38 months, which is longer than the 2.9 months and 3.4 months reported in studies on household transmission in the African meningitis belt (27, 28), but shorter than the 9.4 months observed among school-aged children in Belgium (29). This finding is close to the result of the University of Nottingham study, in which 45% of carriers maintained the same clone for at least 5–6 months (6). A study in Nepal indicated that the maximum Nm carriage duration among household members of IMD patients was ≥60 days (30), while a study in the Netherlands focusing on individuals aged 13–23 years showed that carriage could persist for ≥8 months (31). Furthermore, each serogroup in the present study exhibited a considerable potential for prolonged carriage, with the longest carriage duration being ≥9 months for serogroups B and X, indicating that asymptomatic carriers may serve as potential sources of infection for extended periods. Notably, differences in carriage duration were observed among serogroups; for example, serogroup B had a maximum carriage duration of ≥12 months, and non-groupable strains had a maximum carriage duration of ≥21 months (32). In summary, Nm carriage typically lasts for several months. The study population was characterized by a high carriage rate, long carriage duration, and frequent strain replacement, which may be associated with crowded conditions, high physical training intensity at the boarding martial arts school, and the fact that most students were aged 10–15 years, with immune systems that are not yet fully mature.

In this study, both the risk of Nm acquisition and the risk of Nm clearance were higher among students aged ≥15 years than among those aged <15 years, suggesting that individuals in this age group are more prone to repeated carriage. These findings are consistent with previous studies. McMillan et al. reported that adolescents aged 16 years (OR = 1.97, 95% CI: 1.00–3.86) and those aged ≥17 years (OR = 3.04, 95% CI: 0.97–9.54) were at higher risk of repeated Nm carriage than individuals aged ≤15 years (21), suggesting that older adolescents are at increased risk of repeated Nm carriage. This phenomenon may be associated with the increased frequency and closeness of social contacts among older adolescents. Moreover, increased sebum secretion during puberty leads to marked proliferation of propionate-producing anaerobes, which results in alterations in the nasopharyngeal microenvironment and promotes Nm colonization (33). In addition, individuals with a history of smoking may have a higher risk of repeated Nm carriage. Previous studies have shown that smoking is significantly associated with a higher carriage rate (34, 35). High smoke exposure induces changes in the respiratory mucosa, thereby reducing the self-cleaning capacity of the pharynx and facilitating Nm colonization. Meanwhile, the inflammatory response and mucosal damage caused by smoking may trigger an immune response, leading to partial clearance of colonized Nm and resulting in recurrent carriage. The present study also observed higher Nm clearance rates among those with a history of fever or antibiotic use in the past week. Antibiotic use is negatively associated with Nm carriage rates (34, 36). A systematic review showed that antibiotics such as ceftriaxone and ciprofloxacin can effectively clear Nm from nasopharyngeal colonization (37). During our follow-up, we noted that most cases of recent fever were due to respiratory tract infections, and the concomitant use of antibiotics or other medications, together with the immune response, may have promoted Nm clearance. Given the high carriage rate at the Dengfeng martial arts school, 96.67% (58/60) of new students received the ACYW135 group meningococcal polysaccharide vaccine upon enrollment. Nevertheless, the carriage rate among new students remained high, suggesting that the quadrivalent meningococcal polysaccharide vaccine had no significant impact on Nm carriage and that its population-level protective effect is limited. Studies have shown that meningococcal polysaccharide conjugate vaccines can effectively reduce nasopharyngeal carriage and transmission of Nm, thereby providing indirect protection through herd immunity (38, 39). Although the ACYW135 group meningococcal polysaccharide conjugate vaccine has been licensed in China, it is primarily used in young children and does not cover adolescents, who currently have high carriage rates. Therefore, there is an urgent need to accelerate the development of meningococcal conjugate vaccines suitable for adolescent populations and to conduct well-designed clinical trials that systematically evaluate their long-term effects on carriage prevalence and disease incidence, thereby further refining immunization strategies for this age group.

This study has several limitations. First, a formal agreement analysis between ctrA and sodC was not performed initially, and the exact number of excluded sodC single-positive samples was not recorded. Second, The cohort of newly enrolled students in this study was restricted to a specific population—students at boarding martial arts schools—and had a relatively small sample size; therefore, the generalizability of the findings regarding carriage duration may be somewhat limited. Third, because relatively few clearance events were observed, the absolute estimates of mean carriage duration should be interpreted with caution. Future large-scale studies with larger sample sizes and more frequent sampling are warranted. In addition, molecular typing of the isolated strains was not performed; thus, it could not be determined whether the strains associated with persistent carriage were the same strain. Further molecular typing studies are therefore needed.

In summary, new entrants in boarding martial arts school exhibited a high risk of Nm carriage, with a prolonged average carriage duration and frequent serogroup switching during carriage episodes. Age ≥15 years and recent fever were associated with enhanced Nm clearance. These findings underscore the need for strengthened health surveillance and behavioural interventions to mitigate the risk of persistent carriage and onward transmission in this congregate setting.

## Data Availability

The raw data supporting the conclusions of this article will be made available by the authors, without undue reservation.

## References

[1] Mikucki A Mccluskey NR Kahler CM. The host-pathogen interactions and epicellular lifestyle of *Neisseria meningitidis*. Front Cell Infect Microbiol. (2022) 12:862935. doi: 10.3389/fcimb.2022.862935, 35531336 PMC9072670

[2] Mbaeyi SA Bozio CH Duffy J Rubin LG Hariri S Stephens DS . Meningococcal vaccination: recommendations of the advisory committee on immunization practices, United States, 2020. MMWR Recomm Rep. (2020) 69:1–41. doi: 10.15585/mmwr.rr6909a1, 33417592 PMC7527029

[3] Olbrich KJ Müller D Schumacher S Beck E Meszaros K Koerber F. Systematic review of invasive meningococcal disease: sequelae and quality of life impact on patients and their caregivers. Infect Dis Ther. (2018) 7:421–38. doi: 10.1007/s40121-018-0213-2, 30267220 PMC6249177

[4] Read RC. *Neisseria meningitidis*; clones, carriage, and disease. Clin Microbiol Infect. (2014) 20:391–5. doi: 10.1111/1469-0691.12647, 24766477

[5] Mokhort H. Multiple linear regression model of meningococcal disease in Ukraine: 1992–2015. Comput Math Methods Med. (2020) 2020:1–7. doi: 10.1155/2020/5105120, 32104202 PMC7036103

[6] Bidmos FA Neal KR Oldfield NJ Turner DPJ Ala'Aldeen DAA Bayliss CD. Persistence, replacement, and rapid clonal expansion of meningococcal carriage isolates in a 2008 university student cohort. J Clin Microbiol. (2011) 49:506–12. doi: 10.1128/JCM.01322-10, 21123536 PMC3043478

[7] Ji YF Yang KC Du BH Li ZY Zhang XX Li J . Epidemiological characteristics of meningococcal meningitis cases and trend of serogroup switching of *Neisseria meningitidis* in Henan province, 1950–2021. Mod Disease Control Prev. (2023) 34:7–13. doi: 10.13515/j.cnki.hnjpm.1006-8414.2023.01.015

[8] Li HM Hang RF Wang MK Tian T Zhang H Xia SL . Epidemiological survey and analysis of epidemic cerebrospinal meningitis in three martial arts schools in Dengfeng city, 2017. Pract Prev Med. (2020) 27:479–81.

[9] Li H. Statistical Learning Methods. 2nd ed. Beijing: Tsinghua University Press (2019).

[10] Lipsitch M Abdullahi O D’Amour A Xie WM Weinberger D Tchetgen ET . Estimating rates of carriage acquisition and clearance and competitive ability for pneumococcal serotypes in Kenya with a Markov transition model. Epidemiology. (2012) 23:510–9. doi: 10.1097/EDE.0b013e31824f2f32 PMC3670084 22441543

[11] Thindwa D Wolter N Pinsent A Carrim M Ojal J Tempia S . Estimating the contribution of HIV-infected adults to household pneumococcal transmission in South Africa, 2016–2018: a hidden Markov modelling study. PLoS Comput Biol. (2021) 17:e1009680. doi: 10.1371/journal.pcbi.1009680, 34941865 PMC8699682

[12] Phiri J Sibale L Mlongoti L Mitole N Kusakala A Khwiya M . Estimating pneumococcal carriage dynamics in adults living with HIV in a mature infant pneumococcal conjugate vaccine programme in Malawi, a modelling study. BMC Med. (2024) 22:419. doi: 10.1186/s12916-024-03631-5, 39334289 PMC11438070

[13] Jackson CH. Multi-state models for panel data: the msm package for R. J Stat Softw. (2011) 38:1–28. doi: 10.18637/jss.v038.i08

[14] Jones E Epstein D García-Mochón L. A procedure for deriving formulas to convert transition rates to probabilities for multistate Markov models. Med Decis Mak. (2017) 37:779–89. doi: 10.1177/0272989X17696997, 28379779 PMC5582645

[15] Bureau A Shiboski S Hughes JP. Applications of continuous time hidden Markov models to the study of misclassified disease outcomes. Stat Med. (2003) 22:441–62. doi: 10.1002/sim.1270, 12529874

[16] Cooper B Lipsitch M. The analysis of hospital infection data using hidden Markov models. Biostatistics. (2004) 5:223–37. doi: 10.1093/biostatistics/5.2.223, 15054027

[17] Jackson CH Sharples LD Thompson SG Duffy SW Couto E. Multistate Markov models for disease progression with classification error. JRSS D. (2003) 52:193–209. doi: 10.1111/1467-9884.00351, 40046247

[18] Powell MJD. The BOBYQA Algorithm for Bound Constrained Optimization without Derivatives. Cambridge: (2009).

[19] Wang X Li F Hang TW Cheng ZZ Li Q Li HM . Carriage rate and antibody levels before and after meningococcal vaccine immunization among healthy students in a school in Dengfeng City in 2018. Jiangsu J Prev Med. (2019) 30:430–2. doi: 10.13668/j.issn.1006-9070.2019.04.023

[20] Neal KR Nguyen-Van-Tam JS Jeffrey N Slack RCB Madeley RJ Ait-Tahar K . Changing carriage rate of *Neisseria meningitidis* among university students during the first week of term: cross sectional study. BMJ. (2000) 320:846–9. doi: 10.1136/bmj.320.7238.846, 10731181 PMC27326

[21] Mcmillan M Mohammed H Bednarz J Leong LEX Lawrence A Sullivan TR . Longitudinal study of meningococcal carriage in adolescents and young adults in South Australia 2017–2020. J Infect. (2024) 88:149–57. doi: 10.1016/j.jinf.2024.01.002, 38242365

[22] Gilca R De Wals P Nolan SM Kitchin N Eiden JJ Jiang Q . A longitudinal epidemiology study of meningococcal carriage in students 13–25 years old in Quebec. mSphere. (2018) 3:e00427-18. doi: 10.1128/mSphere.00427-18, 30518675 PMC6282008

[23] Luo JJ Lü J Li TT Xiao J. Analysis of surveillance results of *Neisseria meningitidis* carriage rate among healthy population in Hubei Province, 2013–2018. Appl Prev Med. (2021) 27:180–2.

[24] Zhou JJ Li MS Zhang Q Yan TT Wu D Li YX . Analysis on incidence trend of meningococcal meningitis and major pathogenic serogroups of *Neisseria meningitidis* in China, 1990–2023. Chin J Epidemiol. (2024) 45:1197–203. doi: 10.3760/cma.j.cn112338-20240312-00117, 39307691

[25] Parikh SR Andrews NJ Beebeejaun K Campbell H Ribeiro S Ward C . Effectiveness and impact of a reduced infant schedule of 4CMenB vaccine against group B meningococcal disease in England: a national observational cohort study. Lancet. (2016) 388:2775–82. doi: 10.1016/S0140-6736(16)31921-3, 28100432

[26] Caugant DA Tzanakaki G Kriz P. Lessons from meningococcal carriage studies. FEMS Microbiol Rev. (2007) 31:52–63. doi: 10.1111/j.1574-6976.2006.00052.x, 17233635

[27] Basta NE Berthe A Keita M Onwuchekwa U Tamboura B Traore A . Meningococcal carriage within households in the African meningitis belt: a longitudinal pilot study. J Infect. (2018) 76:140–8. doi: 10.1016/j.jinf.2017.11.006, 29197599 PMC5790055

[28] Consortium M. Household transmission of *Neisseria meningitidis* in the African meningitis belt: a longitudinal cohort study. Lancet Glob Health. (2016) 4:e989–95. doi: 10.1016/S2214-109X(16)30244-3, 27855873

[29] De Wals P Gilquin C De Maeyer S Bouckaert A Noel A Lechat MF . Longitudinal study of asymptomatic meningococcal carriage in two Belgian populations of schoolchildren. J Infect. (1983) 6:147–56. doi: 10.1016/S0163-4453(83)92756-1, 6875288

[30] Sharma S Acharya J Caugant DA Aryal S Banjara MR Ghimire P . Meningococcal carriage among household contacts of patients with invasive meningococcal disease in Kathmandu, Nepal: a longitudinal study. Pathogens. (2021) 10:781. doi: 10.3390/pathogens10070781, 34206153 PMC8308540

[31] Van Ravenhorst MB Bijlsma MW Van Houten MA Struben VMD Anderson AS Eiden J . Meningococcal carriage in Dutch adolescents and young adults; a cross-sectional and longitudinal cohort study. Clin Microbiol Infect. (2017) 23:573.e1–7. doi: 10.1016/j.cmi.2017.02.008, 28192234

[32] Kristinsdottir I Visser LJ Miellet WR Mariman R Pluister G Haraldsson G . Meningococcal carriage in children and young adults: a cross-sectional and longitudinal study, Iceland, 2019–2021. Euro Surveill. (2023) 28:2300215. doi: 10.2807/1560-7917.ES.2023.28.39.2300215, 37768562 PMC10540516

[33] Moir JWB. Meningitis in adolescents: the role of commensal microbiota. Trends Microbiol. (2015) 23:181–2. doi: 10.1016/j.tim.2015.02.004, 25818619

[34] Assefa S Abdissa A Alemu Y Girma L Sahlu D. *Neisseria meningitidis* carriage rate, antibiotic susceptibility profile, and associated factors among prisoners at Jimma zonal correction facility in Jimma town, southwestern Ethiopia: a cross-sectional study. Trop Med Health. (2022) 50:67. doi: 10.1186/s41182-022-00462-z, 36114580 PMC9479249

[35] Tefera Z Mekonnen F Tiruneh M Belachew T. Carriage rate of *Neisseria meningitidis*, antibiotic susceptibility pattern and associated risk factors among primary school children in Gondar town, Northwest Ethiopia. BMC Infect Dis. (2020) 20:358. doi: 10.1186/s12879-020-05080-w, 32434569 PMC7238561

[36] Kizil MC Kilic O Ceyhan M Iseri Nepesov M Karbuz A Kurugol Z . Nasopharyngeal meningococcal carriage among children and adolescents in Turkey in 2018: an unexpected high serogroup X carriage. Children. (2018) 8:871. doi: 10.3390/children8100871, 34682136 PMC8534370

[37] Zalmanovici Trestioreanu A Fraser A Gafter-Gvili A Paul M Leibovici L. Antibiotics for preventing meningococcal infections. Cochrane Database Syst Rev. (2013) 10. doi: 10.1002/14651858.CD004785.pub5, 24163051 PMC6698485

[38] Xu J Yan DS Xu L Li J Shao ZJ. Research progress in global meningococcal polysaccharide conjugate vaccine. Chin J Microbiol Immunol. (2024) 44:304–16. doi: 10.3760/cma.j.cn112309-20231229-00198

[39] Trotter CL Maiden MC. Meningococcal vaccines and herd immunity: lessons learned from serogroup C conjugate vaccination programs. Expert Rev Vaccines. (2009) 8:851–61. doi: 10.1586/erv.09.48, 19538112 PMC3988355

